# Darwin and Neuroscience: The German Connection

**DOI:** 10.3389/fnana.2019.00033

**Published:** 2019-03-26

**Authors:** Gabriel Finkelstein

**Affiliations:** Department of History, University of Colorado Denver, Denver, CO, United States

**Keywords:** Darwin, Emil du Bois-Reymond, Lucretius, Germany, history, nineteenth-century, neuroscience

## Introduction

Ernst Mayr, a leading contributor to the modern evolutionary synthesis, once wrote that “the only solid support Darwin received for natural selection was from the naturalists” (Mayr, 1982, p. 511). Mayr was wrong. The first German convert to Darwin's theory was the neurophysiologist Emil du Bois-Reymond (1818–1896). Why this was so is the subject of this essay.

Du Bois-Reymond had always ridiculed arguments from theological design and organic development (du Bois-Reymond, 1848–1884, p. xxv-l; du Bois-Reymond, 1918, p. 18, 52, 108). The only explanations he deemed scientific were those that dispensed with the “superstition” of final causes (du Bois-Reymond, 1853). This was the main advantage offered by Darwin's theory: it accounted for “the apparently teleological arrangement of nature” without having to rely on either divine intervention or innate purposes (du Bois-Reymond, 1880, p. 75). The principle of natural selection operated mechanically, setting natural history on the same basis as physiology. As du Bois-Reymond saw it, Darwin had done for species what he had done for nerves.

## The First German Darwinist

Du Bois-Reymond's conversion to Darwinism was quick. He initially learned of the publication of the *Origin of Species* from his friend Henry Bence Jones on 21 October 1859. “Darwin's book is expected with great interest,” the English physician wrote to him from London, “and it will be highly unorthodox it is said. It is to be out next month” (Bence Jones, 1859). Du Bois-Reymond was unable to obtain a first edition of the work, but he bought a copy of the second that he read over spring break. “Darwin's book has not told among our scientific men,” he replied to Bence Jones in April. “They consider it a slight aberration of intellect, a heterodoxy painful to look at in an otherwise deserving man” (du Bois-Reymond, 1860a). For his part du Bois-Reymond was convinced. In July he sent Darwin one of his speeches (du Bois-Reymond, 1860b). In November he expressed his “strong approbation” for the theory to a man he knew to be married to Charles Lyell's sister (Darwin, 1860a,b). The next summer he helped Darwin obtain an honorary degree from the University of Breslau (Bence Jones, 1861). And to popularize the *Origin* he compiled a set of popular lectures on the “Findings of Contemporary Science.” First offered in the winter of 1861, du Bois-Reymond's course of lectures regarded the world from a mechanical perspective, one that highlighted the conservation of energy and the theory of evolution. In two consecutive lessons, he reviewed the struggle for existence, natural and sexual selection, and the divergence of varieties in an order where “every niche was filled.” As he described it, Darwin's theory offered five advantages over other conceptions of nature: it provided evidence of ancestry in a common type; it clarified the classification of similar species; it explained the purposeful modification of homologous anatomy; it accounted for the adaptation of organisms to climate and environment; and, most important of all, it eliminated “at one stroke all justification for the suspenseful agony of teleology” (du Bois-Reymond, 1864, p. 19r−19v; du Bois-Reymond, 1912b, p.75; Finkelstein, 2013, p. 245). Du Bois-Reymond's grasp of Darwinian logic was firm.

Du Bois-Reymond taught his course on the “Findings of Contemporary Science” every year over the next three-and-a-half decades. His survey drew on a broad reading of German, French, and English and covered everything from solar flares and flying fish to poison arrows and foot binding. “No one who ever heard Professor du Bois-Reymond of the University of Berlin give his Monday evening lectures on the evolution of scientific thought,” Nicholas Murray Butler recalled as president of Columbia University, “can ever forget the impression which they made upon him nor can he overestimate their value as an instructive and permanent element in his education” (Butler, 1937, p. 341–342). Demand filled the largest auditorium, with listeners spilling into the aisles and crowding the back of the hall. By one estimate up to 10 percent of the university's students—about 800 at the greatest count—attended these spectacles. Many would arrive an hour early to reserve their seats, even if this meant sitting through a class another lecturer had scheduled to capture the audience (Anon, 1896; Finkelstein, 2013, p. 176).

Du Bois-Reymond went on to deliver expositions of the Darwinian theory to large audiences in the Ruhr and the Rhineland (du Bois-Reymond, 1877, 1880; Finkelstein, 2013, p 246). These performances, combined with addresses on Darwin before the Prussian Academy of Sciences, demonstrate his commitment to making evolution known to a broad swathe of scholars, students, and laypeople. Ernst Haeckel may have outsold du Bois-Reymond, but he didn't outshine him as a champion of Darwinism.

Why, then, has du Bois-Reymond been overlooked by historians of evolution? I can think of three answers to this conundrum. The first is the obliteration of du Bois-Reymond's memory. Unlike Charles Darwin and Claude Bernard, who remain heroes in England and France, Emil du Bois-Reymond is generally forgotten in Germany—no streets bear his name, no stamps portray his image, no celebrations are held in his honor, and none of his writings remain in print. Most Germans have never heard of him, and if they have, they generally assume that he was Swiss.

Then there is the association of evolution with a trend of German thought that Ernst Cassirer once described as “the primacy of history” (Cassirer, 1950, p 170). Some historians favor this link between Darwinism and development, as in Mario di Gregorio and Robert Richards's interpretation of Haeckel as a Romantic idealist (Di Gregorio, 2005; Richards, 2008). Other historians disparage it, as in Peter Bowler's dismissal of the “non-Darwinian character of Haeckel's evolutionism” (Bowler, 1988, p. 83–84). Still other historians merely point out the “metaparadigm” of morphological change in German biology (Levit and Hoßfeld, 2017). Whichever view we endorse, du Bois-Reymond's emphasis on natural selection tends to get overlooked. Evolution in the German context becomes a narrative of the parallels between genesis and growth, not a discussion of the problems of variation, inheritance, and adaptation.

Finally, there's the issue of Darwin's distrust. In 1866 Bence Jones invited du Bois-Reymond to deliver a series of lectures at the Royal Institution. Realizing that his famous patient was also scheduled to visit him in London, Bence Jones arranged a meeting between the two scientists. Du Bois-Reymond described Darwin as “a tall man with a high, bald forehead, a long, white beard, friendly, clever eyes, and an extremely kind manner. He volunteered little but questioned me thoroughly. Bence Jones appears to have cured him of his imaginary complaints, from which many people here, Miss Martineau for example, seem to fall deathly ill only in order to revive” (Darwin, 1866; du Bois-Reymond, 1866). But despite the light tone of his irony, du Bois-Reymond's disappointment with the interview was obvious. He had hoped to win Darwin's favor; instead, the English scientist preferred to correspond with Ernst Haeckel (Browne, 1995–2002, p. 269–271). That we remember the German naturalist more than the German neuroscientist has much to do with Darwin's fear of being overshadowed.

## Lucretian Origins

Du Bois-Reymond's father, a Swiss immigrant from the canton of Neuchâtel, clung to Calvinism through the various upheavals of his life. As a young man Emil du Bois-Reymond rebelled against his father's fatalism and embraced *Naturphilosophie*, but before long he came to reject the speculative excesses of this brand of Romantic idealism. What prompted him to change his mind was *De rerum natura* (du Bois-Reymond, 1838; Kiel, 1838).

Lucretius's epic poem had circulated as a defense of the system of Epicurus against the teachings of the Church ever since Poggio Bracciolini discovered it in 1417. In positing a cosmos of matter in motion, devoid of supernatural influence or divine purpose, the work persuaded du Bois-Reymond to abandon the language of animal spirits and investigate nerves with the instruments of physics. In 1843 he succeeded in detecting the action current, or nerve signal, in living organisms; on 3 August 1846, before witnesses at the Berlin Physical Society, he demonstrated the same signal in his own body, revealing the effect of the will to be an electrical phenomenon (du Bois-Reymond, 1846; Finkelstein, 2015).

During the long interval between 1841, when he began his studies of neuroscience, and 1858, when he was appointed Professor of Physiology at the University of Berlin, du Bois-Reymond sought the patronage of Alexander von Humboldt. The famous naturalist had conducted his own experiments on animal electricity, and he took a keen interest in his protégé. Du Bois-Reymond returned the favor by correcting French drafts of Humboldt's *Cosmos*, a four-volume “sketch of the physical description of the universe” (Rupke, 1997, p. 1: vi). In private du Bois-Reymond agreed with Bence Jones (1852) that the Romanticism of Humboldt's outlook was “antediluvian both in mind and matter”; still, his patron's success in presenting a unified vision of nature nagged at his ambition to do the same.

*On the Origin of Species* appeared the year that Humboldt died. Du Bois-Reymond welcomed the book like a debt paid. To his eyes the main attraction of Darwin's theory was the principle of natural selection. Other naturalists had devised systems of transmutation, but only Darwin had imagined evolution as blind (Temkin, 1977, p. 414). That insight allowed du Bois-Reymond to extend his Lucretian vision of nature from the laboratory to the field, recasting the vitalism of *Cosmos* in the idiom of necessity (Finkelstein, 2013, p. 248). “Final causes in nature are incompatible with its intelligibility,” du Bois-Reymond announced in 1876. “Hence, if there is any way of banishing teleology from nature, the scientist has to take it. A way is found in the theory of natural selection…. In holding fast to this theory, we may feel like a man clinging to a plank that only barely keeps him afloat. When the choice lies between a plank and going under, the advantage is decidedly on the side of the plank” (du Bois-Reymond, 1912a, p. 557). Du Bois-Reymond's allusion to Lucretius was clear: the *Origin of Species* may have been a shipwreck, but it was better than foundering with the Romantics or the theologians (Blumenberg, 1997, p. 73–75). Nature was best understood as a mechanical process.

## Author Contributions

The author confirms being the sole contributor of this work and has approved it for publication.

### Conflict of Interest Statement

The author declares that the research was conducted in the absence of any commercial or financial relationships that could be construed as a potential conflict of interest.
